# Histone Chaperones Spt6 and FACT: Similarities and Differences in Modes of Action at Transcribed Genes

**DOI:** 10.4061/2011/625210

**Published:** 2011-10-26

**Authors:** Andrea A. Duina

**Affiliations:** Biology Department, Hendrix College, 1600 Washington Avenue, Conway, AR 72032, USA

## Abstract

The process of gene transcription requires the participation of a large number of factors that collectively promote the accurate and efficient expression of an organism's genetic information. In eukaryotic cells, a subset of these factors can control the chromatin environments across the regulatory and transcribed units of genes to modulate the transcription process and to ensure that the underlying genetic information is utilized properly. This article focuses on two such factors—the highly conserved histone chaperones Spt6 and FACT—that play critical roles in managing chromatin during the gene transcription process. These factors have related but distinct functions during transcription and several recent studies have provided exciting new insights into their mechanisms of action at transcribed genes. A discussion of their respective roles in regulating gene transcription, including their shared and unique contributions to this process, is presented.

## 1. Introduction

In eukaryotic cells, gene transcription takes place in the context of chromatin, a protein-DNA structure that includes the nucleosome—a particle composed of DNA and core histone proteins—as its fundamental unit [1]. The presence of nucleosomes over the regulatory and transcribed regions of genes poses unique problems not encountered by prokaryotic organisms and, as a result, eukaryotic cells have evolved sophisticated mechanisms that enable them to manipulate nucleosomes in a manner that allows for efficient transcriptional control. Different classes of protein factors that can modulate chromatin environments at transcribed loci have been identified and include chromatin remodeling complexes, which can alter DNA-histone interactions in an ATP-dependent manner, histone modifying enzymes, which can modulate the properties of nucleosomes by controlling the set of posttranslational modifications present on the histone proteins within nucleosomes, and histone chaperones, which can interact specifically with histones and can promote the assembly and/or disassembly of nucleosomes in an ATP-independent fashion [2–4]. Several histone chaperones with established roles in transcription have been identified and their contribution to this process is a current area of intense research [3, 5]. This review focuses on two of the better characterized histone chaperones—Spt6 and FACT—and summarizes our current understanding of their roles in the modulation of gene transcription. These highly conserved histone chaperones contribute to the transcription process in several ways and a discussion of the similarities and differences in their mechanisms of action is presented.

## 2. Identification and Initial Characterization of Spt6 and FACT

### 2.1. Spt6

*Spt6* was originally identified in the suppressor of Ty (Spt) screens and selection experiments in *Saccharomyces cerevisiae*, which were designed to identify genes that when mutated or expressed at abnormal levels could suppress the deleterious effects of insertions of Ty and solo **δ** elements at certain biosynthetic genes [6, 7]. Soon after its initial discovery, it became clear that Spt6 plays essential roles in the control of transcription in yeast and that its role was likely to be genome-wide and not confined to regulatory aspects in the context of Ty and **δ** elements [8–13]. Subsequent genetic and biochemical experiments further showed that Spt6 can interact directly with histones (with a preference for histone H3), that it possesses nucleosome assembly activity and that it is an important player in the transcription elongation process [14–16]. These early experiments pointed to critical roles for Spt6 in the control of transcription through interactions with chromatin and provided the foundations for subsequent studies that have shed further light on the cellular processes impacted by Spt6 and the mechanistic aspects of its activities.

### 2.2. FACT

Similarly to Spt6, the components of the FACT (FAcilitates Chromatin Transcription) complex were first isolated through genetic and biochemical approaches in the *S. cerevisiae *model system. The gene encoding the first component of the yeast FACT (yFACT) complex, Spt16 (also known as Cdc68), was isolated as an *SPT *gene based on its ability to suppress the transcriptional defects of **δ** element insertions at the *LYS2 *and *HIS4 *genes when expressed from a high copy number plasmid and was shown to encode an essential protein involved in transcription regulation of several genes [17–21]. The second component of yFACT, Pob3, was originally isolated as a protein that copurifies with DNA Polymerase **α** in biochemical experiments [22] and later shown to also have roles in transcription [23]. Subsequent studies showed that Spt16 and Pob3 form a heterodimer involved in a variety of chromatin-based activities, likely through its ability to interact directly with nucleosomes with the assistance of Nhp6, a protein containing a DNA-binding region similar to the evolutionarily conserved high mobility group (HMG) motif found in several chromatin-interacting proteins [24, 25]. In the literature, the term yFACT has been used to refer to either the Spt16-Pob3 heterodimer or the Spt16-Pob3 dimer in association with Nhp6; for the purpose of this review, the term yFACT will refer to just the Spt16-Pob3 heterodimer, but it is important to keep in mind that the activities ascribed to yFACT are thought to require the participation of Nhp6 as well. The human FACT complex was identified in independent biochemical experiments as an activity required for productive transcription elongation on chromatin templates in *in vitro *reconstitution experiments [26, 27]. These landmark experiments provided critical initial insights into the biochemical properties for which the FACT complex is named. The human FACT complex comprises the homolog of Spt16 (hSpt16) and SSRP1, a protein that combines features of both the yeast Pob3 and Nhp6 proteins, suggesting that during evolution the functions conferred by Pob3 and Nhp6 in yeast have been condensed into a single polypeptide [28].

## 3. Histone Chaperoning and Transcription Regulation by Spt6 and FACT

### 3.1. Spt6

The initial discovery of the ability of Spt6 to interact with histones and to assemble nucleosomes *in vitro *[14] foreshadowed the now well-established role for Spt6 as a key histone chaperone during the transcription process. During transcription elongation, Spt6 is required for the maintenance of a chromatin structure that prevents improper usage of cryptic promoter elements, suggesting that the ability of Spt6 to reassemble nucleosomes in the wake of Pol II passage is critical for the prevention of spurious intragenic transcription initiation [29, 30]. The involvement of Spt6 in proper chromatin reconstitution during transcription elongation is also observed at certain activated stress genes [31, 32]. The requirement for Spt6 to reassemble nucleosomes during Pol II elongation, however, does not apply to all genes but instead appears to be associated predominantly with genes that are transcribed at high rates [33]. These findings are consistent with other studies that have proposed that the fate of nucleosomes during transcription elongation depends on the rate of transcription: in the context of high levels of transcription nucleosomes are completely dismantled in front of Pol II and reconstituted in its wake [34–36] whereas in the context of low levels of transcription hexamers devoid of an H2A-H2B dimer remain associated with DNA while still allowing for Pol II passage through a mechanism involving the formation of small DNA loops [35–37]. Thus, it is possible that the chaperoning activity of Spt6 is required in the former case but not in the latter [33], which would be consistent with the fact that Spt6 is not believed to be a histone H2A-H2B chaperone. Interestingly, however, even in the context of low transcription rates during which nucleosome loss is not detected in an *spt6 *mutant, Spt6 function can still be required to prevent cryptic intragenic transcription, thus pointing to functions for Spt6 in preventing intragenic transcription that are, at least in some cases, independent from its classical role as a histone chaperone during the transcription elongation process [33].

The chaperoning activity of Spt6 is also required for proper control of transcription initiation. Recent studies in yeast have indicated that nucleosome reassembly activity driven by Spt6 occurs over certain gene promoters and that this activity is required for proper transcriptional repression [33, 38]. The observation that the expression of many genes is affected by an *spt6 *mutation [30] but that these genes do not significantly overlap with a set of genes that suffer detectable loss of nucleosomes over their transcribed regions in the same *spt6* mutant background argues that Spt6-driven histone chaperoning activity during transcription elongation does not strongly impact the gene expression process [33]. Therefore, proper transcriptional output likely relies on Spt6-mediated functions that are related to its activity in transcription initiation and/or on Spt6-directed nucleosome reassembly-independent activities during transcription elongation. Finally, Spt6, as well as yFACT, has also been shown to regulate transcription initiation through their histone chaperoning activities during transcription elongation: in this case, transcription of intergenic noncoding DNA that overlaps the promoter of the yeast *SER3 *gene is accompanied by Spt6- and yFACT-dependent reassembly of nucleosomes, which, in turn, are thought to prevent binding of transcription activators required for *SER3 *expression, ultimately resulting in transcription repression of *SER3 *[39].

### 3.2. FACT

The histone-chaperoning activity of the FACT complex during transcription elongation is required for two distinct, but potentially mechanistically related, processes: facilitation of histone removal in front of elongating Pol II and nucleosome reassembly in the wake of Pol II passage. Evidence for the former process has come principally from *in vitro* experiments that showed that (i) FACT can interact with nucleosomes and its activity is required for efficient transcription elongation on nucleosomal templates, (ii) the two subunits of FACT can interact with H2A-H2B dimers and (H3-H4)_2_ tetramers, and (iii) FACT activity can promote loss of H2A-H2B dimers from nucleosomes [26, 27, 40]. Additional evidence in support of this notion has come from recent experiments showing that mutations predicted to weaken interactions between H2A-H2B dimers and (H3-H4)_2_ tetramers can suppress defects in yFACT function both *in vivo *and *in vitro* [41]**—**thus, histone mutations that favor nucleosome disassembly decrease the dependency on yFACT activity, a finding consistent with a role for FACT in promoting histone eviction. Interestingly, additional studies have shown that yFACT activity is required principally at genes that contain stable nucleosomes positioned over the 5′ end of their coding units, suggesting that FACT-mediated histone eviction at the early stages of transcription elongation is a particularly important event for ensuring proper Pol II progression throughout the length of a transcribed gene [42]. Whereas in some instances it has been speculated that the Spt6 histone chaperone may also possess nucleosome-disruption activity, no direct evidence for such an activity has been reported**—**therefore, facilitation of histone removal during the transcription elongation process may represent a major difference in the activities of FACT and Spt6.

Similarly to Spt6, the FACT complex has critical roles in the reassembly of nucleosomes following passage of Pol II over transcribed units. Initial evidence for a role for FACT in transcription-dependent nucleosome reassembly came from experiments in yeast showing synthetic lethal genetic interactions between *spt16* mutants and mutations in factors involved in deposition of histones onto DNA [43]. Whereas FACT was originally categorized as a histone H2A-H2B chaperone based on its ability to interact with H2A-H2B dimers but not with H3-H4 tetramers *in vitro *[27], subsequent biochemical experiments showed that human FACT can deposit all four core histones onto DNA *in vitro,* suggesting that part of FACT's *in vivo* function may include participation in nucleosome reassembly during transcription elongation through interactions with all core histones [40]. Recent experiments have provided support for the ability of FACT to interact with histones H3 and H4 and have highlighted the importance of these interactions in promoting histone deposition onto DNA during transcription elongation [34, 44–46]. Moreover, an elegant set of studies has shown that yFACT functions by incorporating the preexisting histones H3 and H4 back onto DNA following Pol II passage, a process with clear implications for the importance of maintenance of epigenetic marks on core histones over transcribed genes [46]. Recent work has implicated the Spt16-M domain, a structural domain originally identified through partial proteolysis experiments [47, 48], in directing histone deposition during transcription elongation [49]. Collectively, these findings establish FACT as a key chaperone for all four core histones during transcription elongation.

What are the consequences of defective FACT-mediated nucleosome reassembly during transcription elongation? Given the shared functions in transcription-dependent chromatin reassembly with Spt6, it is not surprising that mutations in FACT can also result in cryptic transcription initiation defects [29, 30, 49, 50]. However, unlike the case for Spt6 described earlier in which its histone deposition activity does not appear to be required at infrequently transcribed genes, loss of function of the Spt16 component of yFACT does result in nucleosome loss over certain infrequently transcribed genes and even genes expected to be in the “off” state [46]. Thus, it would appear that, at least in certain instances, infrequently and marginally transcribed genes can undergo nucleosome loss and that reassembly of proper nucleosome structure in these cases depends on FACT but not on Spt6, although one cannot exclude the possibility that the differential requirement observed for the two histone chaperones in this context could be due, at least in part, to the different experimental methodologies used in the two studies that addressed this issue [33, 46]. The requirement for FACT but not for Spt6 in nucleosome reassembly over the bodies of infrequently transcribed genes could be explained by a model in which for this class of genes loss of Spt6-mediated histone chaperoning can be compensated by FACT, which can chaperone all four core histones onto DNA whereas loss of FACT activity cannot be compensated by Spt6, which can only chaperone histones H3 and H4. An extension of this model would be that at highly transcribed genes, due to a demand for rapid and/or frequent nucleosome reassembly, the activities of both Spt6 and FACT become essential for maintenance of proper chromatin structure and loss of either one cannot be compensated by the other. Further genetic and biochemical experiments will need to be carried out to test the validity of this model.

An additional consequence of loss of FACT chaperoning activity has been recently described by Chávez and colleagues. In these studies, a failure of yFACT to properly deposit histones during transcription elongation was shown to lead to abnormally high intracellular levels of free histones, which, in turn, led to a delay in cell cycle progression at the G1 phase by repressing expression of a G1-cyclin gene [51]. Therefore, FACT chaperoning activity is critical both for events directly related to chromatin structure at sites where transcriptional elongation is occurring and, in a more indirect fashion, for proper progression through the cell cycle by controlling the proportion of nucleosomal versus nonnucleosomal histones in the cell. Interestingly, a mutation in *SPT6* was also shown to cause phenotypes consistent with excess accumulation of free histones in cells [51], thus raising the possibility that maintenance of proper levels of free histones in cells is a general property shared with other members of the histone chaperone family.

As is the case for Spt6, FACT activity is also required for proper regulation of transcription initiation. Whereas, as described earlier, Spt6 is required for repression of transcription initiation through its ability to directly promote nucleosome reassembly over gene promoter regions [33, 38], FACT-mediated chromatin alterations, including promotion of histone H2A-H2B removal from nucleosomes, have been implicated in activation of transcription initiation at a variety of genes in a number of different species [52–59]. Thus, FACT and Spt6 can play opposite roles at gene promoters, but both functions are consistent with some of their known biochemical activities—facilitation of histone removal for the FACT complex and reassembly of nucleosomes for Spt6. However, at least in the context of the *SER3 *gene as described earlier, yFACT and Spt6 can both repress transcription initiation through their transcription elongation-dependent nucleosome reassembly activities [39].

## 4. Mechanisms of Spt6 and FACT Histone Chaperoning Activity

### 4.1. Spt6

The mechanistic details for the interactions that occur between Spt6 and nucleosomes during the chaperoning process are still under investigation, but early and more recent studies have shown that Spt6 can bind double-stranded DNA *in vitro *[60] as well as free histones and nucleosomes and that the interaction between Spt6 and intact nucleosomes requires Nhp6, the same HMG protein described earlier that is also required for interactions between nucleosomes and yFACT [14, 61]. The interaction between Spt6 and nucleosomes is also regulated by the Spt6 binding partner Spn1/Iws1. Structural studies have identified a region located toward the N-terminus of Spt6 and a region located toward the C-terminus of Spn1/Iws1 containing two ARM repeats as being responsible for mediating the Spt6-Spn1/Iws1 interaction and functional studies have indicated that the integrity of this interface is critical for the proper function of the complex [61–63]. Interestingly, binding of Spn1/Iws1 to Spt6 interferes with the ability of Spt6 to interact with nucleosomes [61], thus suggesting that *in vivo *Spn1/Iws1 may assist Spt6 in releasing itself from nucleosomes following nucleosome reassembly. Together, the interactions observed between Spt6 and histones, nucleosomes and naked DNA are likely to represent snapshots of a series of events that normally occur during the Spt6-mediated nucleosome reassembly process in the context of gene transcription.

### 4.2. FACT

Similarly to Spt6, the yFACT complex requires the assistance of Nhp6 in order to bind to nucleosomes *in vitro* [25]. A series of elegant studies carried out by Formosa and colleagues has shown that several Nhp6 proteins are required to recruit yFACT to nucleosomes and that significant nucleosomal alterations occur upon Nhp6-mediated yFACT binding to nucleosomes [64, 65]. A major question that is still a subject of debate in the field is whether removal of H2A-H2B dimers from nucleosomes is a direct and necessary result of FACT activity or simply one of several potential outcomes [66]. Whereas the original model for FACT activity, which has been referred to as the “dimer eviction model,” includes a direct role for the complex in dissociation of single histone H2A-H2B dimers from nucleosomes [66, 67], a more recently presented model, which has been referred to as the “global accessibility/noneviction model,” proposes that interaction of FACT with nucleosomes results in the formation of reorganized nucleosomes in which all histone subunits are still tethered together but are in a dynamic structural state more prone to histone H2A-H2B loss [66, 68, 69]. In this latter model, histone H2A-H2B dimer loss from nucleosomes is not a necessary consequence of FACT activity but it is one that can be favored by extrinsic factors such as the force exerted by an oncoming Pol II complex. 

Regardless of the exact mechanism, efficient FACT-facilitated eviction of histones during transcription elongation likely requires specific posttranslational histone modifications. In particular, monoubiquitination of histone H2B (H2BK123ub1 in yeast and H2BK120ub1 in mammals) has been shown to prime nucleosomes for FACT-mediated H2A-H2B dimer loss [70]. In addition, several histone acetyl transferase (HAT) complexes have been implicated as positive factors for transcription elongation (e.g., see [71–73]), with one of them, NuA3, having been shown to interact physically and genetically with the FACT complex [71]. Therefore, various histone modifications are likely to play important roles in regulating the efficacy of FACT in histone eviction during transcription elongation *in vivo* and future research will undoubtedly shed more light on the mechanistic details of these processes.

## 5. Functional Relationships between Spt6 and FACT and Histone Modifications

### 5.1. Spt6

Both Spt6 and FACT have the ability to influence the chromatin environment across transcribed genes by affecting histone posttranslational modifications. Spt6 activity has recently been linked to methylation of lysine 36 of histone H3 (H3K36me), a modification catalyzed by the Set2 histone methyltransferase associated with the reestablishment of proper nucleosome structure in the wake of Pol II passage through the recruitment of the Rpd3S histone deacetylase complex and subsequent histone deacetylation [74–76]. In the yeast system, a specific mutation in Spt6 leads to reduction in both dimethylation and trimethylation of H3K36 (H3K36me2 and H3K36me3, resp.); however, only the H3K36me3 modification appears to be directly promoted by Spt6 as the reduction in H3K36me2 in the *spt6 *mutant appears to be due to an indirect effect resulting from decreased levels of the Set2 protein in the *spt6* mutant background [77, 78]. Interestingly, whereas H3K36me2 has been shown to be required for the prevention of cryptic intragenic transcription initiation through the Rpd3S pathway, H3K36me3 does not appear to be involved in this pathway, thus pointing to roles for Spt6 and Spt6-dependent H3K36me3 in transcription elongation independent from maintenance of proper chromatin structure [78]. A possible role for the Spt6-H3K36me3 pathway has come from studies in mammalian cells. These studies have indicated the existence of a complex bound to elongating Pol II containing Spt6, Iws1, and the HYPB/Set2 histone methyltransferase, which in mammalian cells catalyzes the H3K36me3 modification, and have shown that knockdown of HYPB/Set2 results in accumulation of bulk poly(A)^+^ mRNA in the nucleus [79]. Thus, Spt6 and its partner Iws1 may promote HYPB/Set2-mediated H3K36me3 to facilitate mRNA nuclear export through a mechanism that has yet to be clearly defined.

### 5.2. FACT

As indicated earlier, monoubiquitination of histone H2B facilitates FACT-mediated histone eviction during transcription elongation. Interestingly, *in vitro *and *in vivo *experiments have also demonstrated a requirement for FACT activity in promoting H2B monoubiquitination, a role for H2B monoubiquitination in maintenance of FACT at transcribed regions and a cooperative relationship between H2B monoubiquitination and FACT activity in reassembling nucleosomes during transcription elongation [70, 80]. Thus, these studies establish a positive and dynamic relationship between H2B monoubiquitination and FACT activity during transcription elongation. As a testament to the versatility of histone modifications, recent work has shown that monoubiquitination of histone H2A, in stark contrast to histone H2B monoubiquitination, negatively impacts the process of transcription elongation by inhibiting FACT recruitment to chromatin [81]. These findings set the stage for additional studies exploring the potential interplay between additional histone modifications and FACT activity during transcription elongation.

## 6. Roles for Spt6 and FACT in mRNA Processing and Nuclear Export

In addition to being key contributors to the initiation and elongation phases of transcription, Spt6 and FACT are also involved in functions related to mRNA processing and nuclear export. In mammalian cells, the Pol II-associated complex discussed earlier composed of Spt6, Iws1, and HYPB/Set2 plays important roles in ensuring proper mRNA splicing and efficient mRNA export from the nucleus [79, 82]. The FACT complex has also been shown to participate in the process of mRNA nuclear export [83, 84], with recent experiments showing a direct interaction between the SSRP component of FACT and the mRNA export adaptor UIF [84]. Moreover, experiments performed in yeast have shown that Spt6 can regulate site selection for transcription termination and mRNA 3′ end formation [85, 86] and both Spt6 and Spt16 are required for efficient RNA splicing [87]. Taken together, these findings establish Spt6 and FACT as important players in the processes of mRNA processing and nuclear export and provide insights into the mechanisms that ensure the coordinated execution of the different phases that ultimately lead to the proper formation and localization of mRNA molecules. 

## 7. Mechanisms of Spt6 and FACT Interactions with Transcribed Genes

### 7.1. Spt6

Pioneering immunofluorescence and biochemical experiments performed in *S. cerevisiae *and *D. melanogaster* provided compelling evidence that, as had been anticipated based on its characteristics as an elongation factor [15], Spt6 physically associates with Pol II and that it interacts with chromatin following patterns of interaction similar to those seen for transcribing Pol II [88–90]. More recent studies have provided additional insights into both the pattern of association of Spt6 across transcribed genes as well as the mechanisms that control its recruitment and association with chromatin.

Chromatin immunoprecipitation (ChIP) assays in yeast have shown that Spt6 interacts with several constitutively expressed genes in a manner similar to that seen for Pol II [91]. Similar results were obtained in genome-wide experiments in yeast that describe Spt6 as a component of a general transcription elongation complex acting at all transcribed genes [92]. In a recent study by the Lis laboratory in which the recruitment of Pol II and several transcription elongation factors were analyzed using a system that allows for a high degree of temporal resolution, Spt6 was shown to associate with the *Hsp70 *loci in flies upon heat-shock treatment a few seconds after the recruitment of Pol II to the promoter [93]. Collectively, these experiments establish Spt6 as a general transcription elongation factor that is recruited to activated genes shortly after Pol II recruitment and that travels across transcribed units likely in association with the Pol II complex.

What are the mechanisms that control Spt6 association with transcribed genes? A critical function involved in directing Spt6 interaction with the elongating complex is carried out by a tandem SH2 (tSH2) domain located at the C-terminus of the protein. Analyses of recently solved crystal structures of the Spt6 tSH2 domain derived from different organisms have shown that the overall structure of this domain is evolutionarily conserved and a series of biochemical experiments has shown that this domain mediates interactions between Spt6 and the C-terminal domain (CTD) heptad repeats of Pol II [60, 82, 94, 95]. The interaction between the Spt6 tSH2 domain and the Pol II CTD is direct and requires phosphorylated serine residues on the CTD—more specifically, Ser2-phosphorylation on the CTD appears to be generally required for this interaction [60, 79, 82, 94, 95] whereas an involvement for Ser5-phosphorylation on the CTD, either by itself or in combination with Ser2-phosphorylation, in directing this interaction is less clear since conflicting results have been reported on this issue, likely as a consequence of differences in the assays and/or model systems used in the different studies [60, 79, 82, 94]. Interestingly, mammalian Spt6 is able to discriminate between different regions of the mammalian Pol II CTD and shows specific interactions with the N-terminal half of the CTD [79]. Additional properties of the tSH2 domain of Spt6 have been revealed through fluorescence anisotropy experiments, which have shown that the Spt6 tSH2 domain can also bind to CTD peptides that had been artificially phosphorylated on tyrosine residues present at the first position of the CTD heptad repeats, indicating that the tSH2 domain of Spt6 has phosphotyrosine-binding activity—which is the activity normally associated with SH2 motifs present in certain higher eukaryotic proteins—and raising the intriguing possibility that Spt6 may also specifically bind to target proteins through more canonical SH2-phosphotyrosine interactions [60].

Whereas the direct interaction between the tSH2 domain of Spt6 and Pol II clearly contributes to the association of Spt6 with transcribed genes, it is not the sole mechanism involved in recruitment of Spt6 to active genes. In support of this notion, a mutation within the SH2 domain of murine Spt6 that lowers the affinity of Spt6 to Ser2-phoshporylated Pol II CTD did not affect transcription output levels in either *in vitro* or *in vivo *assays [82]. Furthermore, Mayer et al. have shown that in yeast the pattern of Pol II CTD Ser-2 phosphorylation across transcribed genes does not correlate with Spt6 occupancy and, more importantly, have reported that a mutant version of Spt6 that lacks the tSH2 domain can still be recruited to the 5′ ends of genes, albeit to a lesser degree than what is seen with wild-type Spt6 [92]. The fact that the Spt6 tSH2 domain is required for optimal recruitment of Spt6 to transcribed genes in these latter experiments suggests that the Spt6 tSH2-Pol II CTD interaction plays a role in the initial recruitment of Spt6 to chromatin: this recruitment may involve interactions between the Spt6 tSH2 and Ser5-phosphorylated versions of the Pol II CTD (which have been reported to occur *in vitro *[60]) or may be mediated through Ser-2 phosphorylated Pol II CTD, which, albeit present at low levels at 5′ ends of genes, could nevertheless recruit Spt6 since, at least in the context of the *Drosophila *Hsp70 genes, arrival of the Ser2 Pol II CTD kinase P-TEFb precedes Spt6 recruitment [93]. In addition to the role in Spt6 recruitment to transcribed genes, the interaction between Spt6 and Pol II mediated by the Spt6 tSH2 and the Ser2-phosphorylated Pol II CTD has been shown to play critical roles in regulating mRNA processing and nuclear export in mammals through Spt6-dependent recruitment of Iws1 and additional factors to nascent RNA molecules as discussed earlier [79, 82].

**Figure 1:**
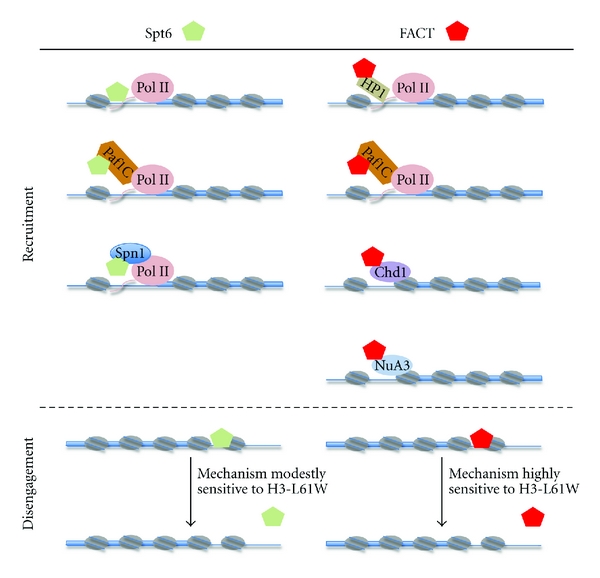
Proposed models for Spt6 and FACT recruitment to and disengagement from actively transcribing genes. *Recruitment (top panel): *Several mechanisms for Spt6 recruitment to active genes have been proposed and include direct interactions with the Pol II CTD (tail extending from Pol II in the figure) and indirect interactions with Pol II through either the Paf1 complex (Paf1C in the figure) or Spn1/Iws1 (Spn1 in the figure) serving as bridging factors. The FACT complex is thought to be able to associate indirectly with Pol II with either HP1 or the Paf1 complex bridging the interaction. The chromatin remodeling factor Chd1 and the histone acetyl transferase complex NuA3 can interact with histones and with FACT and likely promote FACT association with chromatin. *Disengagement (bottom panel):* at some genes, Spt6 and FACT dissociate at distinct locations during the transcription process and, therefore, in these cases each factor must utilize a unique dissociation mechanism (not addressed in this figure). At certain other genes, such as the yeast *PMA1 *and *ADH1* genes, Spt6 and FACT depart chromatin at similar locations. However, the nature of the mechanisms used by the two histone chaperones at this class of genes is likely to be at least to some degree different, with Spt6 using a mechanism that is only modestly sensitive to the H3-L61W mutation and FACT using a mechanism that is very sensitive to the H3-L61W mutation. Green pentagons: Spt6; red pentagons: FACT; thin blue lines: DNA regions flanking the coding region of a transcribed gene; thick blue lines: coding region of a transcribed gene; gray ovals with two blue lines: nucleosomes.

The observation that deletion of the Spt6 tSH2 domain does not abolish recruitment of Spt6 to transcribed genes indicates that additional mechanisms must exist to ensure proper Spt6 recruitment to 5′ ends of genes. Experiments performed in *Drosophila *and in *S. cerevisiae *provide some insights into the nature of these mechanisms. In *Drosophila*, impairment of the Paf1 complex—a multifunctional complex associated with Pol II that coordinates a variety of transcription-related processes, including recruitment of several transcription factors to genes and various posttranslational modifications of histones [96]—results in lower levels of Spt6 occupancy at the* Hsp70* gene [97]. Whereas the Paf1 complex has been shown to be required for full levels of Ser2 phosphorylation of the Pol II CTD in certain contexts [98, 99], the decrease in Spt6 association at the *Drosophila Hsp70 *gene does not appear to be an indirect effect due to lower CTD phosphorylation since at this locus the levels of Ser2-phosphorylation of the Pol II CTD are not affected by the depletion of the Paf1 complex [97]. Thus, at least in certain cases, the Paf1 complex appears to be involved in recruitment of Spt6 to transcribed genes in a manner independent from its role in regulating Pol II modifications. An alternative mode of recruitment of Spt6 to a transcribing gene has been described for the yeast *CYC1 *gene. In this case, Spt6 recruitment is dependent on Spn1/Iws1 [100], which has been shown to possess roles in transcription regulation downstream from initial recruitment of the TATA-box binding protein (TBP) to gene promoters [90, 101, 102]. Whether this latter recruitment mechanism is widespread in the yeast genome or is limited to those genes that are regulated at a post-TBP and post-Pol II recruitment step (as is the case for the *CYC1 *gene) remains to be more fully elucidated (see Figure 1 for a cartoon depiction of the proposed mechanisms for Spt6 recruitment to active genes).

### 7.2. FACT

Several lines of evidence have demonstrated that the FACT complex, similarly to Spt6, physically associates with the bodies of transcribed genes *in vivo*. Numerous ChIP studies in yeast have shown specific interactions between yFACT and the transcribed regions of several actively transcribing genes [50, 91, 92, 103] and immunofluorescence and ChIP experiments in *Drosophila* have shown that FACT colocalizes with hyperphosphorylated Pol II at many transcriptionally active loci [104]. Interestingly, these latter experiments also showed that the patterns of association of FACT, Spt6, and Pol II at the activated heat shock gene *hsp70* are similar to one another [104], consistent with the notion that the two histone chaperones function in conjunction with each other to assist Pol II during the transcription elongation process. Comparative ChIP studies in yeast have provided further support for this notion since at certain active genes Spt6, yFACT, and Pol II associate with chromatin following similar patterns as one another [91, 105]. The hypothesis that FACT and Spt6 operate in conjunction with each other and with elongating Pol II during transcription elongation is also supported by several reports that have shown that the two chaperones can be found in the same physical complexes that also contain hyperphosphorylated Pol II [102, 104]. However, FACT and Spt6 are also likely to have roles independent from each other in transcription elongation since a recent genome-wide study has shown that at the global level, yFACT and Spt6 associate with an “average” gene in overlapping but distinct patterns, in which yFACT appears to be recruited at a location slightly more upstream from that used by Spt6 and is released earlier in the elongation process than Spt6 [92]. Thus, whereas it is likely that FACT and Spt6 can operate in conjunction during transcription elongation, their functions do not appear to be always coordinated with each other.

How is the FACT complex recruited to the transcribed regions of genes? Several studies have provided support for a role for the ATP-dependent chromatin remodeling factor Chd1 in recruitment of FACT to actively transcribing genes. Mammalian Chd1 and FACT interact physically with each other and in *Drosophila* they display similar patterns of association across polytene chromosomes [106]. Similarly, experiments carried out in yeast have shown that Chd1 associates with transcribed regions of active genes and that it can be found in complexes containing components of the yFACT complex [90, 107]. Since human Chd1 can associate with histone H3 methylated at lysine 4 (H3K4me3)—a histone modification associated with actively transcribed genes—through its two chromodomains [108–110], it is possible that at least in certain cases Chd1 can direct FACT recruitment to chromatin at sites enriched for the H3K4me3 modification [67, 111]. Strongly supporting this possibility, human FACT and Chd1 can be copurified with H3K4me3-containing peptides with Chd1 being responsible for bridging the histone H3 and FACT interaction [112]. The generality of this model, however, is unclear since recent studies have shown that unlike its human counterpart, yeast Chd1 does not bind to histone H3 peptides methylated at lysine 4 [109, 110, 113]. The relationship between Chd1 and FACT is further complicated by the finding that, at least in certain cases, Chd1 and Spt16 can have opposing functions during transcription [56]. Thus, whereas it is likely that at least in higher eukaryotes Chd1 can directly recruit FACT to sites of active transcription through its ability to interact with H3K4me3-containing nucleosomes, Chd1 and FACT also display additional types of functional interactions that still remain to be more fully elucidated.

FACT recruitment to active genes is also controlled by the Paf1 complex. Evidence in support of this notion includes experiments carried out in yeast that have revealed physical and genetic interactions between components of the Paf1 complex and FACT [90, 114, 115], and studies in flies showing that depletion of Paf1 complex components decreases recruitment of FACT to the activated *Hsp70 *gene [97]. As indicated earlier, these latter experiments also showed a requirement for the Paf1 complex in the association of Spt6 to chromatin, thus establishing a potential common route for Spt6 and FACT recruitment to actively transcribing genes. However, a physical relationship between Spt6 and the Paf1 complex has not been as clearly defined in the yeast model system; thus, whether the requirement for the Paf1 complex in efficient recruitment of Spt6 to the *Hsp70* gene in flies reflects a broader physical and functional connection between the two factors remains to be more thoroughly investigated. In addition to directly recruiting FACT to sites of active transcription, the Paf1 complex may also facilitate FACT recruitment through indirect mechanisms stemming from its ability to modulate histone modifications—for example, it could be envisioned that the Paf1 complex localized to genes via direct interactions with Pol II leads to Paf1 complex-dependent histone modifications, which include H2B monoubiquitination and subsequent histone H3K4me3, which in turn recruits Chd1, ultimately leading to FACT association. The existence of such a pathway involving the Paf1 complex, Chd1, and FACT is consistent with experiments performed in yeast indicating that the three factors genetically interact with each other [114]. The H3K4me3 mark may also facilitate recruitment of the FACT complex through the NuA3 histone acetyl transferase complex since NuA3 can be bind directly to H3K4me3, as well as unmodified, histone H3 tails and, as indicated earlier, NuA3 physically associates with FACT [71, 116, 117]. Unlike the case with Spt6, the FACT complex does not appear to directly interact with Pol II; however, recent work in the fly system by Workman and colleagues has provided strong evidence that the FACT complex can interact with phosphorylated Pol II through a bridging protein, the heterochromatin protein 1 (HP1) [118]. In particular, the HP1c isoform is required for optimal recruitment of FACT to several heat shock loci following heat shock treatment and for normal levels of heat shock transcript levels [118]. Collectively, these studies reveal that different types of mechanisms can contribute to FACT recruitment to transcribed regions of genes and they pave the way for additional studies to assess whether all these mechanisms operate in a coordinated fashion at all loci to ensure optimal FACT recruitment or if different sets of recruiting mechanisms are utilized in a gene-specific fashion (see Figure 1 for a cartoon depiction of proposed mechanisms for FACT recruitment to active genes).

## 8. Mechanisms of Spt6 and FACT Disengagement from Transcribed Genes

The mechanisms that control the dissociation of transcription elongation factors, including those with histone chaperoning activity, still remain to be elucidated. It seems reasonable to speculate that at least in some instances the factors that are known to interact with Pol II and that disengage from transcribed units at the same locations as Pol II—that is, downstream from the polyadenylation (pA) sites—may simply dissociate from chromatin in conjunction with the Pol II complex. On the other hand, those factors shown to disengage from transcribed genes at or upstream from the pA sites, such as the Paf1 complex [91, 92], must use different mechanisms for dissociation, which may include alterations in Pol II CTD phosphorylation patterns, competition for Pol II binding with other transcription elongation factors—particularly those that are recruited towards the 3′ end of genes, such as Elf1 and termination factors [91, 92]—and conformational changes of Pol II and associated factors that may occur during the elongation process. 

Whereas, as indicated earlier, global studies in yeast have indicated that at an “average” gene Spt6 and yFACT disengage from chromatin at different sites [92], suggesting that the two factors normally utilize different mechanisms of chromatin dissociation, at some loci, such as at the yeast *PMA1 *and *ADH1 *genes, Spt6 and yFACT disengage at similar locations past the pA sites [91, 103, 105] raising the possibility that at these genes Spt6 and yFACT may utilize similar dissociation mechanisms. An insight into this possibility was recently obtained from experiments in which a histone H3 mutant, H3-L61W, was shown to cause a marked accumulation of yFACT at the 3′ ends of transcribed genes in a transcription-dependent fashion [103, 105]. These studies led to a model in which normally yFACT requires a signal, possibly through posttranslational modification of one or more histone proteins, in order to properly dissociate from chromatin following the transcription process, and that the H3-L61W mutation interferes with this signal by preventing either the proper initiation or the propagation of the signal [103]. Interestingly, the same histone H3 mutation was shown not to significantly affect Pol II departure from *PMA1* and to only modestly affect the dissociation of Spt6 from the *PMA1 *and *ADH1* genes [103, 105]. Therefore, it appears that even at genes in which Spt6 and yFACT normally disengage from chromatin at the same location, Spt6 and yFACT use distinct mechanisms for chromatin dissociation, with yFACT using an H3-L61W-sensitive mechanism and Spt6 using a mechanism that is significantly less sensitive to the H3-L61W mutation [105]. The Spt16-M domain appears to play a role in controlling yFACT dissociation from chromatin since specific mutations in this region have been shown to alleviate the yFACT 3′ accumulation defect seen in H3-L61W cells [49, 103]. Additional studies will be needed to obtain a more complete understanding of the mechanisms that govern dissociation of FACT and Spt6 from chromatin at the end of the transcription process (see Figure 1 for a cartoon depiction of the proposed mechanisms for Spt6 and FACT disengagement from active genes).

## 9. Conclusion

Whereas our understanding of the roles and the mechanisms of action of both Spt6 and FACT in the transcription process has increased dramatically since their original identification more than two decades ago, many questions remain to be addressed and future research will undoubtedly provide a more complete picture of the mechanistic details of the transcription process in general, and, more specifically, of the contributions of these two key histone chaperones to this process. In particular, it will be of interest to determine how the functions of Spt6 and FACT are coordinated with those of other histone chaperones known to operate during transcription, such as Asf1 and Nap1 [3, 5]. Traditional genetic and biochemical approaches coupled with more recent genome-wide strategies will continue to provide a powerful experimental platform with which to address these and other relevant questions.
